# The relationship between childhood socioeconomic status and depression level in older adults: the mediating role of adult socioeconomic status and subjective well-being

**DOI:** 10.1186/s12877-024-04750-7

**Published:** 2024-02-07

**Authors:** Yulin Chai, Guowei Xian, Lin Guo, Guoqi Fu, Yanxu Liu, Mengxue Wang, Sheng Luo

**Affiliations:** School of Management, Shandong Second Medical University, Weifang, Shandong 261053 China

**Keywords:** Socioeconomic status, Older adults, Depression, Subjective well-being

## Abstract

**Background:**

There is a causal link between childhood socioeconomic status and health status in adulthood and beyond. It's vital to comprehend the relationship between childhood socioeconomic status and mental health among older Chinese individuals from the current generation who have undergone significant social changes in China. This understanding is critical to foster healthy demographic and social development in China.

**Methods:**

Using data from the 2020 China Family Panel Studies, we investigate the relationship between childhood socioeconomic status and depression in older adults. Additionally, we examine the mediating role of adult socioeconomic status and subjective well-being.

**Results:**

1) Childhood socioeconomic status of Chinese older adults differences by region of residence, while depression levels differences by gender, region of residence, and marital status. 2) Adult socioeconomic status mediated the relationship between childhood socioeconomic status and depression in older adults. 3) Adult socioeconomic status and subjective well-being had a chain-mediated role in the relationship between childhood socioeconomic status and depression in older adults.

**Conclusions:**

In terms of childhood socioeconomic status, older adults in urban regions were significantly higher than those in rural regions. As for depression level, female older adults were more depressed than males; married older people have the lowest depression levels, while unmarried and widowed older people have higher depression levels; older adults in rural regions had higher depression levels than those in urban regions. Evidence from our study further suggests that childhood socioeconomic status can suppress the depression level in older adults through adult socioeconomic status; it can also further reduce the depression level in older adults through the chain mediation of adult economic status affecting subjective well-being. As depression is more prevalent among older individuals with a lower childhood socioeconomic status, it is vital to prioritize the extensive impact of childhood socioeconomic status as a distal factor and investigate "upstream" solutions to enhance childhood socioeconomic status and reduce the gap during the early years of life.

**Supplementary Information:**

The online version contains supplementary material available at 10.1186/s12877-024-04750-7.

## Introduction

The relationship between socioeconomic status and health is a longstanding topic in social medicine. According to [1], as society evolves and the socioeconomic status gap widens, the modern healthcare system becomes more intricate. People are increasingly expected to be actively involved in promoting and maintaining their own health, posing a challenge. Socioeconomic disadvantage has been linked to negative impacts on both physical and mental health, as evidenced by numerous studies [2–4]. An individual's health is largely determined by their life circumstances, including their social environment, access to education and employment, and income level. These factors are commonly referred to as social determinants of health [5]. Health inequities are amplified by unequal socio-economic status, which serves as a fundamental driver of health disparities since socio-economic status reflects an array of pivotal social determinants of health [6].

According to the life course perspective, health is dynamic and related to socioeconomic status throughout the life course; unfavorable socioeconomic status early in life has a permanent and irreversible negative effect on health, but this effect is not fixed; childhood socioeconomic status can lead to poor adult health outcomes, but the significance of these associations varies after adjusting for adult socioeconomic status [7]. Due to the importance of childhood socioeconomic status on later health outcomes, an expanding body of scholars has investigated the causal relationship between the two [8–10]. Additionally, [11] contend that adult socioeconomic status can both reflect one's childhood socioeconomic status and affect their later health status. Childhood socioeconomic status continues to impact the health of older adults even when later-life socioeconomic status is considered [12]. The intricacy of this phenomenon is due to the various potential factors that affect both health and socioeconomic status, as well as the dynamic nature of both throughout one's life course [13].

Research has established a significant correlation between mental and physical health, yet there remains insufficient knowledge regarding the impact of mental health on overall wellness [14]. Nevertheless, mental and substance use disorders have been the foremost cause of global healthy life loss since 2010, with a 41% surge in their burden compared to 1990. Of these, depression was the leading cause, accounting for 40.5% [15, 16]. By 2017, depression had become one of the top four leading causes of morbidity worldwide [17], 2018); and in 2019 depression emerged as one of the two most debilitating mental illnesses and one of the top 25 global disease burdens, with no consistent reduction in its overall burden [18]. Additionally, due to the epidemic's worldwide impact, it is projected that there will be a 27.6% rise in major depressive disorders worldwide in 2020 [19]. In psychology, happiness, well-being, and mental health are frequently used interchangeably [20]. Consequently, scholars have observed that assessing mental health necessitates considering both well-being and mental illness (e.g., depression) [21, 22] investigation revealed a significant association between subjective well-being and mental health.

For rapidly developing China, unique political, economic, cultural, and social transitions between 1945 and present day have had a lasting impact on its socioeconomic structure, leading to disparities in health outcomes at different times. As a consequence, populations born and raised in different historical periods may exhibit significant differences [23]. The current stage of older adults in China has experienced a series of social changes, underscoring the importance of recognizing the link between childhood socioeconomic status and mental health to promote healthy development of China's population and society [24].

Several studies have been conducted in the past to reveal the important impact of childhood experiences on adult health outcomes. [25] argued that childhood maltreatment adversely affects adult health outcomes and that each additional experience of childhood maltreatment is associated with an increased chance of health problems in adulthood, [26] studied older adults in developing countries for the first time, and demonstrated that childhood socioeconomic status can influence health in later life through various channels, [27] explored the correlation between childhood adversity and depression symptoms among middle-aged and older adults in China, with the moderating role of urbanization. Some studies have further explored the relationship between childhood socioeconomic status and later life health and the role of adult socioeconomic status in this. [28] proposed that childhood socioeconomic status has a significant impact on depression in older adults, but that it is partially moderated by adult socioeconomic status and later life socioeconomic status, [29] demonstrated that adult socioeconomic status became a mediator between childhood experiences and health limitations in older adults at age of 65, while at age of 75 only childhood socioeconomic status and adult socioeconomic status were still directly predictive of later life health outcomes,The study by [30] showed more directly that adult socioeconomic status had a significant mediating effect on the relationship between childhood socioeconomic status and later life health. In addition, some studies have shown subjective well-being also has a significant association between childhood experiences and their mental health in adulthood. Positive psychology suggests that depression is not solely a result of negative cognition but also a lack of positive resources. The differences in CSS can influence SWB, and SWB being an important positive resource, can have an impact on DL [31]. A study by [32] showed that subjective well-being fully mediates the relationship between childhood maltreatment experiences and depression conditions in adulthood,and a study by [33] showed that childhood socioeconomic status was the most important for adulthood among childhood experiences for adult health (10.60%) and also that childhood socioeconomic status is most important for subjective well-being (20.60%). However, there is limited research on whether and how adult socioeconomic status and subjective well-being mediate the relationship between childhood socioeconomic status and depression levels in older adults. Therefore, the purpose of this study was to examine the relationship between childhood socioeconomic status and depression levels among older adults and the mediating role of adult socioeconomic status and subjective well-being therein. We proposed the following hypotheses: 1) Depression levels may be higher in older adults with lower childhood socioeconomic status,2) adult socioeconomic status and subjective well-being have a chain mediating role between childhood socioeconomic status and depression levels in older adults.

## Methods

### Data description

**Fig. 1:**
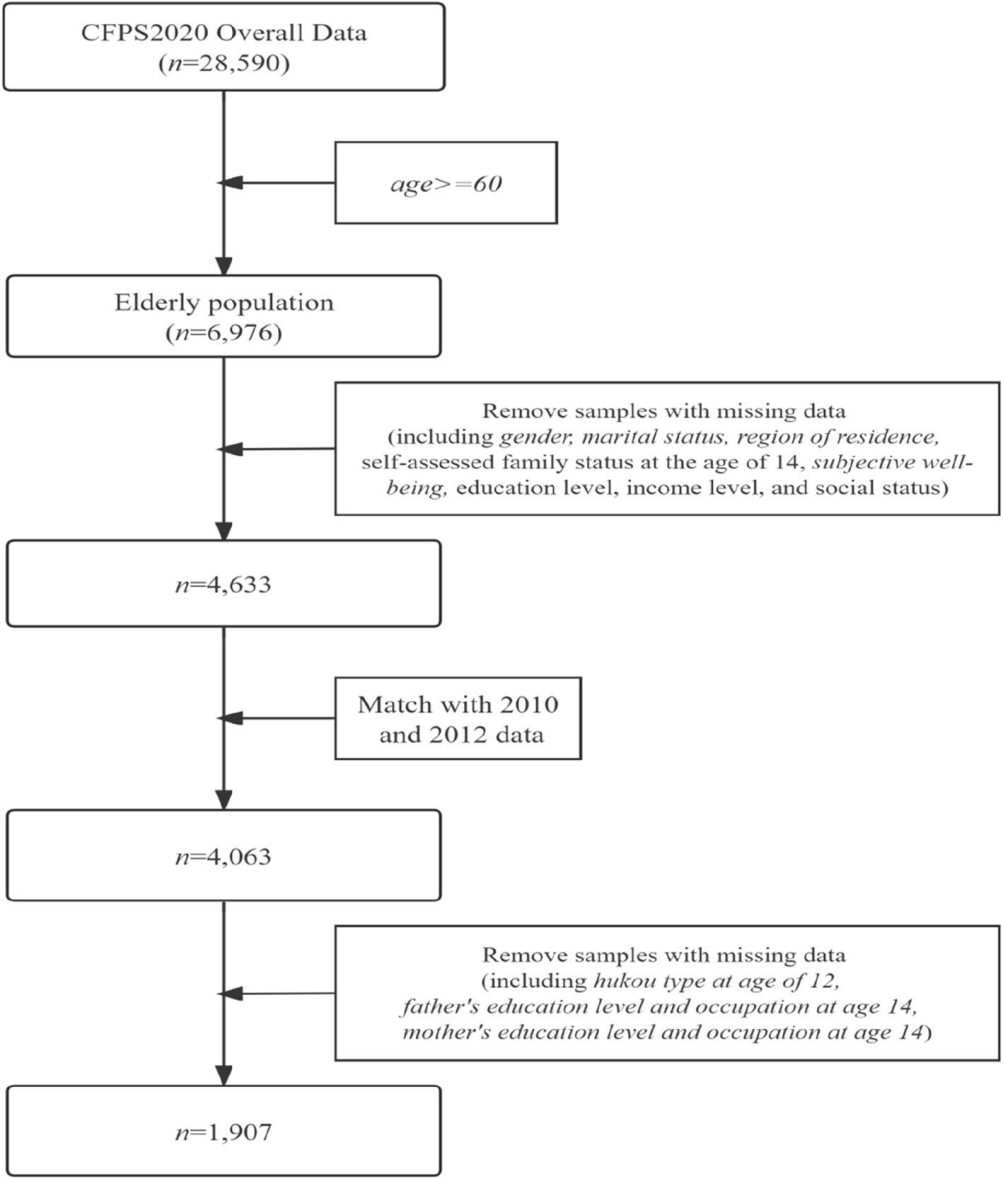
Flowchart for data incorporation

The data for this study come from the China Family Panel Studies (CFPS), a nationwide, large-scale, multidisciplinary social tracking survey program.The CFPS sample covered 25 provinces, with a target sample size of 16,000 households, and included all family members in the sampled households.The final sample size was 14,960 households and 42,590 individuals. This study used the latest CFPS 2020 data, but because it doesn't include enough information on childhood socioeconomic status, we merged the 2010 and 2012 data based on individual IDs. For this study, we selected older adults aged 60 and above, with a final sample size of 1907 and no missing sample data. The screening process is shown in Fig. 1.

## Measures

### Independent variable

The primary independent variable of this study is Childhood Socioeconomic Status (CSS). According to the study of [34], this study selected four variables, including hukou type at the age of 12, self-assessed family status at the age of 14, parents' educational level at the age of 14, and parents' occupational status at the age of 14. In the CFPS, the hukou type at the age of 12 is categorized as non-agricultural or agricultural. The self-assessed family status at the age of 14 is scored on a 5-point Likert scale. The parents' education level at the age of 14 is categorized into 8 levels, ranging from "illiterate/semi-illiterate" to " doctor", and the parents' occupation at the age of 14 is recorded using the ISCO88 code. In this study, Hukou type at the age of 12 was assigned a score of 1 for non-agricultural and 0 for agricultural,self-assessed family status at the age of 14 was assigned original scores ranging from 1 to 5, with higher scores indicating higher childhood family economic status; parents' education level at the age of 14 was assigned scores ranging from 1 to 8 based on eight levels from lowest to highest; and parents' occupational status at the age of 14 was transformed into the International Socioeconomic Index (ISEI) by referring to [35] study, which was assigned a score of 1–4 based on the 25%, 50%, and 75% quartiles of the range of values corresponding to the socioeconomic status of the lower, lower-middle, upper-middle, and upper strata of society, respectively. Finally, we applied principal component analysis to obtain a continuous variable to measure CSS (*KMO* = 0.679, *P* < 0.01).

### Dependent variable

The primary dependent variable of this study is the Depression Level (DL) of older adults, and CFPS used the Center of Epidemiologic Studies—Depression (CES-D) scale to measure the level of depression among study participants. The original CES-D questionnaire contained 20 items, but CFPS employed only 8 of them as the main reference. 1) I felt depressed; 2) I felt that everything I did was an effort; 3) My sleep was restless; 4) I was happy; 5) I felt lonely; 6) I enjoyed life; 7) I felt sad; 8) I could not get "going". Each question has four options: almost never, sometimes, often, and mostly. The four levels are scored as 1, 2, 3, and 4, with questions 4 and 6 being reverse scored. The total score for the eight entries is 24, with higher scores indicating deeper depression.

### Mediator variables

The mediator variables in this study are adult socioeconomic status (ASS) and subjective well-being (SWB). Referring to the study conducted by Luo et al. [36], three variables were selected, specifically education level, income level, and social status. A continuous variable was then obtained through principal component analysis(*KMO* = 0.506, *P* < 0.01). In the CFPS, education level was initially categorized into ten levels, which were consolidated into 6 levels of "Illiterate/Semi-literate" "Elementary school and below" "Junior high school" "High school/Technical school" "Junior college" "Undergraduate and above", each scored from 0 to 5. Participants were asked "How would you rate your income in terms of your location? " and "How would you rate your social status of your location?" which were categorized into 5 levels from very low to very high and scored from 1 to 5. In this study, subjective well-being was also selected as a mediator variable. Due to the limitations of the CFPS and after referring to related studies [21, 37–39], we chose self-assessed well-being as the basis of the SWB measure. In the CFPS, the participants were asked, "How happy do you feel?" Higher subjective well-being is associated with scores ranging from 0 to 10.

### Covariates

The covariates were mainly demographic characteristics including age, gender, marital status, and region of residence. In CFPS, marital status includes five categories: unmarried, married, cohabiting, divorced, and widowed. We combine cohabiting and unmarried according to the marital status specified in the law and ultimately classify them into four categories: unmarried, married, divorced, and widowed. The region of residence is classified into urban and rural according to urban and rural codes.

### Statistical analysis

We analyzed the data using SPSS 25.0. The demographic characteristics were expressed as frequency ( percent) or mean ± SD. We analyzed correlations using Pearson's correlation analysis, and we analyzed differences between groups using ANOVA. Furthermore, we used SPSS PROCESS V4.2 written by Hayes [40] to test the mediating role of the mediator variables between CSS and DL. We considered the differences to be statistically significant at *P* < 0.05.

## Results

### Descriptive statistics

Table 1 shows the descriptive statistics of all variables. The mean age of the 1907 older adults in this study was 67.67 ± 5.51 years; 881 (46.2%) were female and 1026 (53.8%) were male; 23 (1.2%) were unmarried, 1653 (86.7%) were married, 21 (1.1%) were divorced, and 210 (11.0%) were widowed; 1099 (57.6%) resided in rural and 808 (42.4%) in urban; CSS averaged 2.04 ± 0.52 points, ASS averaged 2.19 ± 0.75 points, SWB averaged 7.73 ± 2.12 points, and DL averaged 13.64 ± 4.43 points.

**Table 1:** Descriptive statistics (sample *n* = *1907*)

Variables	Frequency	Percent(%)	CSS		DL		Mean	SD
			Mean ± SD	*P*	Mean ± SD	*P*		
**Age**							67.67	5.51
**Gender**								
Male	1026	53.8	2.03 ± 0.51	0.303	12.84 ± 4.20	< 0.01		
Female	881	46.2	2.05 ± 0.53		14.58 ± 4.51			
**Marriage**								
Unmarried	23	1.2	1.99 ± 0.48	0.512	15.96 ± 4.73	< 0.01		
Married	1653	86.7	2.04 ± 0.52		13.41 ± 4.35			
Divorced	21	1.1	2.05 ± 0.62		14.48 ± 5.32			
Widowed	210	11.0	1.99 ± 0.48		15.15 ± 4.62			
**Residence**								
Rural	1099	57.6	2.01 ± 0.48	0.013	14.19 ± 4.50	< 0.01		
Urban	808	42.4	2.07 ± 0.56		12.89 ± 4.22			
**CSS**							2.04	0.52
**ASS**							2.19	0.75
**SWB**							7.73	2.12
**DL**							13.64	4.43

### Correlation analysis

Appendix 1 shows that CSS was positively correlated with ASS and SWB (*P* < 0.05); ASS was positively correlated with SWB (*P* < 0.05); and DL was negatively correlated with CSS, ASS, and SWB (*P* < 0.05).

## Analysis of mediation effects

Based on the results of descriptive and correlation analyses, this study further examined the possible mediating effects of adult socioeconomic status and subjective well-being between childhood socioeconomic status and the depression level of older adults by using a chain mediation model controlling for age, gender, marriage status, and residence region, and conducted a bootstrap test to verify its significance. Table 2 shows that Table 3 shows that CSS positively predicted ASS (*β* = 0.443, *P* < 0.01); ASS positively predicted SWB (*β* = 0.492, *P* < 0.01) and negatively predicted DL (*β* = -0.828, *P* < 0.01); SWB negatively predicted DL (*β* = -0.571, *P* < 0.01).

**Table 2:** Results of regression analysis of the chain mediation effects model

	ASS				SWB				DL			
	*β*	*SE*	*t*	*p*	*β*	*SE*	*t*	*p*	*β*	*SE*	*t*	*p*
Constant	2.190**	0.205	10.676	< 0.01	4.800**	0.655	7.322	< 0.01	21.321**	1.288	16.551	< 0.01
Age	-0.016**	0.003	-5.536	< 0.01	0.029**	0.009	3.203	0.001	-0.011	0.017	-0.633	0.527
Gender	0.374**	0.031	11.983	< 0.01	-0.114	0.101	-1.129	0.259	-1.319**	0.195	-6.770	< 0.01
Marriage	-0.041	0.025	-1.672	0.095	-0.196*	0.077	-2.555	0.011	0.390**	0.149	2.618	0.009
Residence	0.146**	0.031	4.696	< 0.01	0.084	0.097	0.865	0.387	-1.071**	0.188	-5.685	< 0.01
CSS	0.443**	0.030	14.863	< 0.01	0.182	0.098	1.864	0.063	-0.202	0.190	-1.068	0.286
ASS					0.492**	0.071	6.916	< 0.01	-0.828**	0.140	-5.928	< 0.01
SWB									-0.571**	0.044	-12.832	< 0.01
*R*^*2*^	0.192				0.041				0.175			
*Adjusted R*^*2*^	0.190				0.038				0.172			
*P*	< 0.01				< 0.01				< 0.01			

*P < 0.05 ** *P* < 0.01

**Table 3:** Mediation effect bootstrap test

	Model pathways	Estimate	*SE*	*P*	*95%CI*	percentage
Direct effect	CSS⇒DL	-0.202	0.190	0.286	-0.574,0.169	
Indirect effect	CSS⇒ASS⇒DL	-0.367	0.008	< 0.01	-0.059,-0.028	46.05%
	CSS⇒SWB⇒DL	-0.104	0.007	< 0.01	-0.026,0.001	
	CSS⇒ASS⇒SWB⇒DL	-0.124	0.003	< 0.01	-0.020,-0.010	15.56%
Total indirect effect		-0.595	0.011	< 0.01	-0.091,-0.049	74.65%
Total effect		-0.797	0.190	< 0.01	-1.169,-0.426	100%

Table 3 shows that the direct effect of CSS on DL was not statistically significant (*95% CI* = [-0.574,0.169]), the mediation effect of ASS in CSS and DL was statistically significant (*95% CI* = [-0.574,0.169]), which accounted for 46. 05% of the total effect. The mediation effect of SWB in CSS and DL was not statistically significant (*95% CI* = [-0.026,0.001]). In addition, the chained mediation effect of ASS and SWB in CSS and old DL was statistically significant (*95% CI* = [-0.020,-0.010]), which accounted for 15.56% of the total effect.

**Fig. 2:**
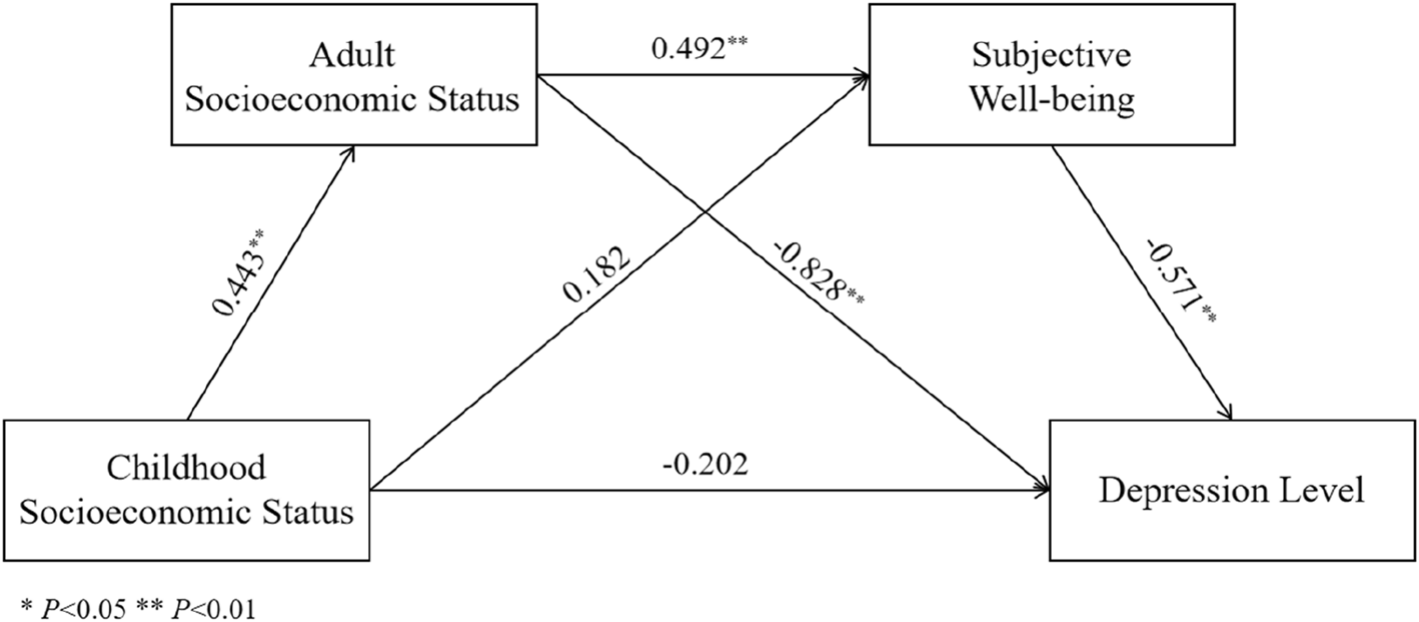
Chain mediation effects model

Finally, we get the chain mediation effects model shown in Fig. 2.

## Discussion

This study's results show that childhood socioeconomic status has significant differences in terms of residence region, with older adults in urban regions being higher than those in rural regions, which is partly consistent with the study by [41]. This might be due to the difference in the level of economic development between urban and rural regions, where rural residents are more different from urban residents in terms of both economic conditions and social status [42]. Moreover, urban regions have more complete health care, social welfare, and cultural and recreational facilities, and it is relatively easier for urban older adults to achieve a better living standard and social status, which ultimately leads to a significantly higher socioeconomic status of urban older adults than rural older adults [43]. Meanwhile, there is a significant difference in the depression level of the older adults in terms of gender, with females being more depressed than males, which is consistent with the study of [44], whose findings verified a higher percentage of depression in females from a genetic perspective. According to the Response Styles Theory (RST), the gender difference in depression level is due to the fact that females tend to ruminate more than males and are more likely to self-reflect and tap into their inner feelings [45], as well as the fact that females go through more physiological changes, such as pregnancy, gestation, etc., and are exposed to more stress in their lives,and are exposed to more social pressures in society, such as family responsibilities, occupational discrimination, etc.,Secondly, there was also a significant difference in the depression level of older adults by marriage status, with the lowest depression level being married, followed by divorced, widowed and the highest being unmarried. These results are similar to the study by Bulloch et al. [46], which found a significant interaction between depression level and marriage status, with those who were divorced or separated being at higher risk of depression. While another study showed that single people feel more lonely, which in turn leads to higher depression level [47]. On the one hand, spouses can provide emotional support and comfort, whereas unmarried or widowed people lack such emotion support and are more likely to feel lonely and lost. On the other hand, couples can support each other and share burdens and stresses, thereby reducing depression,Finally, the depression level of older adults also differs significantly by residence, with rural older adults having higher depression level than urban older adults, which is supported by the study of Yan et al. [48], who showed that depression develops differently in urban and rural regions and that the factors associated with it differ, and that rural older adults are more likely to maintain higher depression level. Rural regions have relatively less social support, more difficult economic conditions, and lower welfare benefits compared to urban regions, all of which can increase the depression of older adults in rural regions.

This study found that adult socioeconomic status can mediate the relationship between childhood socioeconomic status and depression level in older adults, which is supported by studies by Arx et al. [49] and Angelini et al. [50]. Early life environment can have a long-term impact on one's future decisions [51], and socioeconomic status has a certain degree of stability and heritability [52], so that childhood socioeconomic status can influence adult socioeconomic status,and higher socioeconomic status usually means better income, better social security and support, and even better health conditions, among other factors, all of which help to promote mental health and reduce depression level. Many studies have shown that socioeconomic status has long-term effects on physical functioning and is significantly associated with mental health in older adults, and that lower socioeconomic status in childhood increases the risk of depression, stress and anxiety in adulthood [53–56].

This study also found that adult socioeconomic status and subjective well-being chain-mediated the relationship between childhood socioeconomic status and depression level in older adults. Specifically, childhood socioeconomic status positively predicted adult socioeconomic status, and adult socioeconomic status positively predicted subjective well-being, which is partially similar to the study by Wang et al. [57], which believed that adults with higher socioeconomic status were more likely to have positive social mood. The study by Wanberg et al. [58] also showed that individuals with lower socioeconomic status tended to have lower subjective well-being,subjective well-being negatively predicted depression level, which is partially similar to the study by Zhou et al. [37], which showed that subjective well-being is negatively correlated with depression and that people with higher socioeconomic status are significantly happier. A study by Fan et al. [59], explains the relationship between subjective well-being and depression level in genetic terms, showing that people with higher scores for the Serotonin Accumulation Gene tend to have higher subjective well-being and a lower risk of depression.

## Conclusions

In this study, we constructed and tested a chain mediation effect model using cross-sectional data from the CFPS to explain the correlation between childhood socioeconomic status and depression levels among older adults, as well as how adult socioeconomic status and subjective well-being played a mediating role in it. This study partly bridges the gap in this area and provides some theoretical basis for promoting mental health maintenance across the lifecycle.

Our study found that in terms of childhood socioeconomic status, older adults in urban regions were significantly higher than those in rural regions, which may be related to the differences in China's urban and rural levels of economic development and social welfare benefits. As for depression level, female older adults were more depressed than males, which may be related to women's physiological conditions and social pressure. Married older people have the lowest depression levels, while unmarried and widowed older people have higher depression levels, which may be related to the emotional support and stress sharing of spouses. Meanwhile, older adults in rural regions had higher depression levels than those in urban regions, which may be related to poorer social support and economic conditions in rural regions. Evidence from our study further suggests that childhood socioeconomic status can suppress the depression level in older adults through adult socioeconomic status; it can also further reduce the depression level in older adults through the chain mediation of adult economic status affecting subjective well-being.

Our study shows the importance of good childhood socioeconomic status for the mental health of older adults, and in the future, we should pay more attention to the long-term role of childhood as a distal factor, rather than only the current social determinants of health, by focusing on improving children's subjective socioeconomic status from an early age [60]. By focusing 'upstream' in the life courses, improving childhood socioeconomic status and narrowing the gap in early life to avoid cumulative effects, it is possible to achieve a better foundation for health and ensure better health in later life, which is conducive to reducing the risk of depression in old adults.

However, this study has some limitations. The data in this study are cross-sectional, which means it is difficult to establish a clear causal relationship, and future studies could conduct further longitudinal studies based on follow-up data.

### Supplementary Information

**Additional file 1: Appendix 1. **Pearson's correlation coefficient results.** Appendix 2. **Descriptive statistics of original variables for CSS and ASS.

## Data Availability

The CFPS data were obtained from Peking University Open Research Data, available from the official data platform of CFPS (http://www.isss.pku.edu.cn/cfps/download/index#/fileTreeList), and the data in this study were used with permission from CFPS.
