# Supplementary material for: The relationship between childhood socioeconomic status and depression level in older adults: the mediating role of adult socioeconomic status and subjective well-being
Source: BMC Geriatr. 2024 Feb 7;24:138. doi: 10.1186/s12877-024-04750-7 (PMC10848464; doi:10.1186/s12877-024-04750-7)
Supplement: Supplementary file 1 — Additional file 1: Appendix 1. Pearson's correlation coefficient results. Appendix 2. Descriptive statistics of original variables for CSS and ASS. [file 12877_2024_4750_MOESM1_ESM.docx]

# Appendix

**Appendix 1** Pearson's correlation coefficient results

|  | CSS | ASS | SWB | DL |
| --- | --- | --- | --- | --- |
| CSS | 1.000 |  |  |  |
| ASS | 0.317** | 1.000 |  |  |
| SWB | 0.097** | 0.178** | 1.000 |  |
| DL | -0.098** | -0.250** | -0.313** | 1.000 |
| **P*<0.05 ** *P*<0.01 | | | | |

**Appendix 2** Descriptive statistics of original variables for CSS and ASS

| Variables | Frequency | Percent(%) | Mean | SD |
| --- | --- | --- | --- | --- |
|  |  |  |  |  |
| **CSS** |  |  |  |  |
| **Hukou type at the age of 12** |  |  |  |  |
| Agricultural | 1776 | 93.13 |  |  |
| Non-Agricultural | 131 | 6.87 |  |  |
| **Self-assessed family status at the age of 14** |  |  | 3.58 | 1.17 |
| **Father' educational level at the age of 14** |  |  |  |  |
| Illiterate/Semi-literate | 1344 | 70.48 |  |  |
| Elementary school | 412 | 21.60 |  |  |
| Junior high school | 100 | 5.24 |  |  |
| High school/Vocational school/Technical school/Secondary vocational school | 33 | 1.73 |  |  |
| Junior college | 10 | 0.52 |  |  |
| Undergraduate | 8 | 0.42 |  |  |
| Master's degree | 0 | 0.00 |  |  |
| Doctorate | 0 | 0.00 |  |  |
| **Mother' educational level at the age of 14** |  |  |  |  |
| Illiterate/Semi-literate | 1748 | 91.66 |  |  |
| Elementary school | 131 | 6.87 |  |  |
| Junior high school | 20 | 1.05 |  |  |
| High school/Vocational school/Technical school/Secondary vocational school | 6 | 0.31 |  |  |
| Junior college | 0 | 0.00 |  |  |
| Undergraduate | 2 | 0.10 |  |  |
| Master's degree | 0 | 0.00 |  |  |
| Doctorate | 0 | 0.00 |  |  |
| **Father' occupational status at the age of 14** |  |  |  |  |
| Lower | 1626 | 85.26 |  |  |
| Lower-middle | 34 | 1.78 |  |  |
| Upper-middle | 37 | 1.94 |  |  |
| Upper | 210 | 11.01 |  |  |
| **Mother' occupational status at the age of 14** |  |  |  |  |
| Lower | 1818 | 95.33 |  |  |
| Lower-middle | 28 | 1.47 |  |  |
| Upper-middle | 24 | 1.26 |  |  |
| Upper | 37 | 1.94 |  |  |
| **ASS** |  |  |  |  |
| **Education level** |  |  |  |  |
| Illiterate/Semi-literate | 822 | 43.10 |  |  |
| Elementary school and below | 412 | 21.60 |  |  |
| Junior high school | 388 | 20.35 |  |  |
| High school/Technical school | 239 | 12.53 |  |  |
| Junior college | 39 | 2.05 |  |  |
| Undergraduate and above | 7 | 0.37 |  |  |
| **Income level** |  |  | 3.55 | 1.06 |
| **Social status** |  |  | 3.23 | 1.14 |
